# Association of Estimated Total Body Iron with All-Cause Mortality in Japanese Hemodialysis Patients: The Miyazaki Dialysis Cohort Study

**DOI:** 10.3390/nu15214658

**Published:** 2023-11-03

**Authors:** Tatsunori Toida, Yuji Sato, Hiroyuki Komatsu, Shouichi Fujimoto

**Affiliations:** 1 School of Pharmaceutical Sciences, Kyushu University of Health and Welfare, Nobeoka City 882-8508, Miyazaki, Japan; 2 Department of Internal Medicine, Division of Nephrology, National Health Insurance Takachiho Town Hospital, Takachiho 889-1101, Miyazaki, Japan; ysato@med.miyazaki-u.ac.jp; 3 Center for Medical Education and Career Development, Faculty of Medicine, University of Miyazaki, Miyazaki City 889-16095, Miyazaki, Japan; hiroyuki_komatsu@med.miyazaki-u.ac.jp; 4 Department of Medical Environment Innovation, Faculty of Medicine, University of Miyazaki, Miyazaki City 889-1609, Miyazaki, Japan; fujimos@med.miyazaki-u.ac.jp

**Keywords:** anemia, iron deficiency, total body iron, hemodialysis, mortality

## Abstract

Iron deficiency/excess may be associated with worse prognosis in patients undergoing hemodialysis. This study ascertained the association of the estimated total body iron (TBI) with mortality in patients receiving hemodialysis. Multicenter clinical data collected in the Miyazaki Dialysis Cohort Study from 943 patients receiving hemodialysis were analyzed after stratification into tertile categories by baseline TBI—estimated as the heme iron plus iron storage from ferritin levels. The primary outcome was a 5-year all-cause mortality; hazard ratios of the TBI–all-cause mortality association were estimated using Cox models adjusted for potential confounders, including clinical characteristics, laboratory, and drug data, wherein patients with high TBI were the reference category. The receiver operating characteristic (ROC) curve analyses of TBI, serum ferritin levels, and transferrin saturation were performed to predict all-cause mortality; a total of 232 patients died during the follow-up. The low TBI group (<1.6 g) had significantly higher hazard ratios of mortality than the high TBI group (≥2.0 g). As ROC curve analyses showed, TBI predicted mortality more accurately than either levels of serum ferritin or transferrin saturation. Lower TBI increases the mortality risk of Japanese hemodialysis patients, and further studies should examine whether iron supplementation therapy that avoids low TBI improves prognosis.

## 1. Introduction

Iron, a vital microelement for most cells, is essential in several biological processes, such as erythropoiesis [1]. Currently, there is no well-established laboratory method for diagnosing iron deficiency or excess. As current guideline recommendations suggest, indicators of iron metabolism include transferrin saturation (TSAT) and levels of ferritin [2,3]. However, the levels of serum ferritin fluctuate in several diseases, including inflammatory diseases, infections, liver diseases, and malignancies as well as with intravenous iron administration, and only increase when the effects of erythropoiesis-stimulating agents wear off [4]. Although the levels of serum ferritin may be normal or high, the possibility of impaired utilization for hematopoiesis due to iron maldistribution exists [5]. The use of the TSAT to estimate iron metabolism is complicated because of the following issues. First, it is difficult to accurately determine the level of TSAT because: (1) the TSAT is derived as the serum Fe to total iron-binding capacity ratio, which is dependent on the severity of inflammation and nutritional status [6,7], and (2) the TSAT varies significantly during the day [8,9,10]. Moreover, the TSAT exhibits an acute phase reaction because of the elevated levels of transferrin levels during acute inflammatory processes, resulting in reduced TSAT with constant circulating iron levels [11]. The TSAT and levels of serum ferritin have both shown association with prognosis in patients with chronic kidney disease (CKD) treated with or without hemodialysis (HD); however, the cutoff value for TSAT varies among studies [12,13,14,15]. Furthermore, the serum ferritin level differs among countries and regions [16] and the C-reactive protein level has been identified as an important confounder [17], which also varies widely among countries and regions. Thus, the relationship with ferritin levels needs to be considered [16]. Currently, in clinical settings, in evaluating iron stores (IS) and ascertaining iron availability, the levels of ferritin as well as TSAT are used most commonly as markers. However, as agreed by participants at the recent Kidney Disease: Improving Global Outcomes (KDIGO) conference [18], for estimating body IS or predicting responses to therapy, current parameters are unreliable. The development of new indices for iron deficiency/excess independent of inflammation is necessary.

Therefore, we used a formula for estimating total body iron (TBI) from hemoglobin and ferritin levels [19] and aimed to assess and quantify TBI in patients who are on maintenance HD; furthermore, we investigated the relationship of TBI with all-cause mortality.

## 2. Materials and Methods

### 2.1. Participants

**Figure 1:**
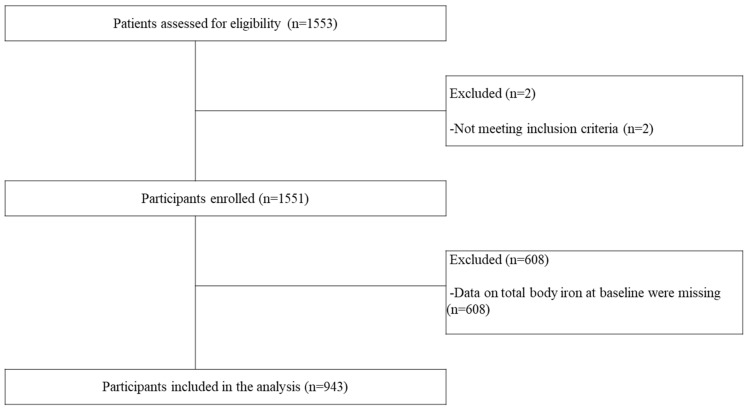
Flowchart of participant screening and selection.

In December 2009, the University of Miyazaki, Japan, initiated a prospective observational study (Miyazaki Dialysis Cohort Study) that included 943 patients on maintenance HD from 27 dialysis centers. The follow-up period of the study was 5 years. The final analysis of this study excluded patients with ≤3 months of dialysis vintage; who were aged <18 years; or who were pregnant, hospitalized, or unwilling to participate. Patients receiving iron supplements/infusions/medications were not excluded (Figure 1).

### 2.2. Extraction of General Clinical Data and Measurement of Laboratory Indices

Information on physical characteristics, laboratory data, basal renal diseases, comorbidities, and medications was collected by doctors in each dialysis center at the start of the study. Blood samples were taken in a supine position pre-HD in the first dialysis session of the week at baseline. Serum albumin-adjusted calcium was calculated as:

Serum calcium (mg/dL) + 4 − serum albumin (g/dL)

When the serum albumin was <4 g/dL. Blood pressure values were averaged from 3 consecutive HD sessions during the week of patient enrollment.

### 2.3. Predictors

The TBI was calculated as follows:

TBI = red cell iron (g) + IS

To measure the levels of red cell iron (i.e., heme iron), we used the estimated value of 3.4 mg iron/g of hemoglobin [20]. Using the Kaplan formula, the estimated blood volume was calculated from the body weight and the hematocrit [21], and red cell iron levels were then evaluated from the estimated blood volume and venous hemoglobin (per L) as [22]:

Estimated blood volume × hemoglobin × 0.91

The IS was estimated using the following formula [2,19]:

IS (mg) = [−13.8588 + 0.3939 × G + 15.5999log_10_(F) − 2.0519(log_10_(F)) (G: male; 1, female; 0, F = ferritin)] × body weight

Furthermore, the IS/TBI ratio was used to investigate the impact of iron distribution on outcomes.

### 2.4. Outcomes

The primary outcome was all-cause mortality during the 5-year follow-up. Survival information (death and cause of death) was verified by medical staff during the monthly follow-up by questionnaires and rechecked by two authors (YS and TT) as relevant. We collected the check sheets annually. Causes of death were categorized as cardiovascular, infective, and other. Cardiovascular mortality included deaths due to ischemic/hemorrhagic stroke, acute myocardial infarction, and causes related to congestive heart failure, sudden death, or aortic aneurysmal rupture. Survival time defined based on the time from enrollment to individual outcomes was derived using the longitudinal data collected until December 2014.

### 2.5. Statistical Analysis

To determine the normality of continuous data, the Kolmogorov–Smirnov test was performed, and normally distributed data were reported as mean ± standard deviation (SD) or median (interquartile range) for non-normally distributed data. Patient characteristics were reported after performing descriptive analyses according to the following three TBI tertile groups: <1.6, 1.6–2.0, and ≥2.0 g. The one-way analysis of variance (ANOVA) and Kruskal–Wallis test were performed to compare normally and non-normally distributed continuous variables, respectively, among the three groups, using a two-tailed test. The Kaplan–Meier survival analysis was performed to determine the probability of survival while the log-rank test was performed to compare the survival curves by registry. To determine the association among TBI categories, IS/TBI ratios, and outcomes, the Cox regression model, adjusted for potential confounders, was developed to estimate hazard ratios (HR). Model 1 was adjusted for age and sex; and model 2 was adjusted for model 1 as well as dry weight, walking independently, smokers, systolic and diastolic blood pressure, time of dialysis therapy, type of vascular access, diabetes mellitus, history of cardiovascular diseases, single-pool Kt/V, hemoglobin, serum albumin, serum C-reactive protein, adjusted serum phosphate and calcium, the use of erythropoiesis-stimulating agents, angiotensin-receptor blockers, and angiotensin-converting enzyme inhibitors. Patients with high TBI and IS/TBI ratios constituted the reference category. The assumption of proportional hazards model, based on the Kaplan–Meier method and log–log plot was met by all the covariates. The method used to deal with the missing covariates was multiple imputation by chained equations. The receiver operating characteristic (ROC) curves were evaluated, and areas under the curves (AUCs) were determined to verify whether TBI, IS/TBI ratios, ferritin, and TSAT were predictors of all-cause mortality. For the statistical analyses, IBM SPSS Statistics for Windows, version 25.0 (IBM Corp., Armonk, NY, USA) and Stata MP 15.0 (Stata Corp, College Station, TX, USA) were used.

### 2.6. Ethical Considerations

The present study fulfilled the condition of the principles of the Declaration of Helsinki and the University of Miyazaki Research Ethics Committee (No. 740) approved the protocol. Data collection procedures upheld the patients’ anonymity principle (UMIN000005160). Verbal informed consent was obtained from all participants and was recorded on patient medical charts by the attending physicians. The University of Miyazaki Research Ethics Committee approved the use of the verbal informed consent modality. Furthermore, the right of the participants to refuse or withdraw from study participation was ensured by the conspicuous placement of a poster announcement to this effect in each of the 27 dialysis clinic or center.

## 3. Results

### 3.1. Baseline Characteristics of Participants

**Figure 2:**
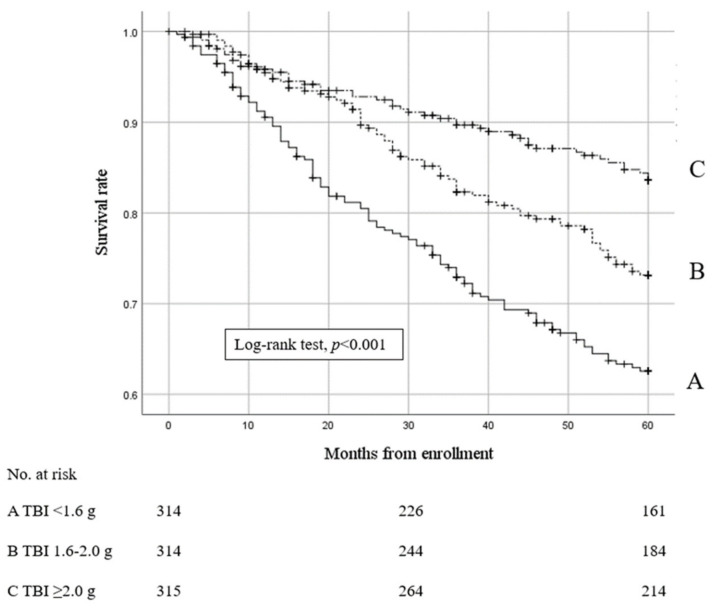
Kaplan–Meier estimates for survival rates among groups stratified via baseline serum total body iron levels.

In total, 943 participants were enrolled in the cohort study (Figure 1). At enrollment, the mean TBI (SD) was 1.9 (0.5) g and had a normal distribution (via the Kolmogorov–Smirnov test, *p* = 0.100; Figure 2). Table 1 shows baseline participant characteristics in the three groups, which were stratified according to baseline TBI. Significant intergroup differences were observed in age, sex, the duration of HD duration, pre-HD diastolic blood pressure, dry weight, inter-dialysis weight gain, diabetes mellitus, walking independently, smokers, anti-hypertensive drug use, hemoglobin, TSAT, serum ferritin, red cell iron, IS, TBI, erythropoietin resistance index (ERI), serum blood urine nitrogen, serum creatinine, serum intact parathyroid hormone, serum albumin, and single-pool Kt/V by ANOVA, the Kruskal–Wallis test, or the χ^2^ test.

### 3.2. Effects of TBI on Mortality

In the 5-year follow-up, 232 patients died of all-cause mortality, including 103 cardiovascular, 52 infections, and 77 other causes; moreover, 151 (16.0%) participants moved to other dialysis facilities, including those who underwent kidney transplantation.

Figure 2 shows that the all-cause mortality rate was significantly higher in the lowest TBI group than in the other TBI groups (Kaplan–Meier analysis, the log–rank test, *p* < 0.001).

Table 2 shows that, in the adjusted analysis of all-cause mortality, HRs were significantly higher in patients with TBI <1.6 g than in the reference group. Furthermore, cardiovascular, infective, and other mortality proportions showed similar results.

### 3.3. Effects of IS/TBI on Mortality

Table 3 shows that, in the adjusted analysis of all-cause mortality, HRs were significantly higher in patients with IS/TBI ≥0.30 than in the reference group. Furthermore, cardiovascular, infective, and other mortalities showed similar results.

### 3.4. Subgroup Analyses

Subgroup analyses of several prespecified variables were conducted, and the adjusted HRs were calculated for all-cause mortality in the highest versus the lowest tertile of TBI (Table 4) and IS/TBI ratios (Table 5). HRs for all-cause and other mortality in the lowest TBI tertile were elevated across the subgroups. No significant interactions were observed for any of the variables. Similar results were obtained as those of IS/TBI.

### 3.5. ROC Curve Analysis

According to the ROC curves of TBI and IS/TBI for predicting all-cause mortality, the AUCs of TBI for predicting all-cause mortality was 0.639 (95% CI 0.599–0.679, *p* < 0.001), whereas that for IS/TBI was 0.592 (95% CI 0.551–0.633, *p* < 0.001). The AUCs of levels of serum ferritin levels and TSAT for predicting all-cause mortality were 0.575 (95% CI 0.534–0.617, *p* < 0.001) and 0.506 (95% CI 0.453–0.559, *p* = 0.831), respectively. 

## 4. Discussion

The present study provided several novel insights. Lower TBI and higher IS/TBI increased the mortality risk. Furthermore, subgroup analyses of several prespecified variables were conducted and revealed that HRs for all-cause mortality in the lowest TBI tertile were elevated across the subgroups. In the ROC curve analysis, the AUCs for TBI and IS/TBI were greater than those for serum ferritin levels and TSAT.

Cable et al. previously reported the estimation of TBI in a general population after whole-blood donation [19]. This approach only focuses on the two major compartments, namely hemoglobin and IS. This measurement did not consider minor iron compartments, like transferrin, myoglobin, and iron cofactors in enzymes and cytochromes, although these are more appropriate in assessing the body’s iron because the minor compartments form a very small proportion in the body’s IS.

Iron plays a crucial role in cell functions and cardiac muscle metabolism [23]. Iron deficiency by itself may directly aggravate the severity of cardiac functional abnormalities and contribute to high mortality independent of anemia [24]. Furthermore, iron deficiency, considered to be TSAT < 20% with high or low serum ferritin levels [11,25], was identified as a predictor of excess hospitalization [26] and higher mortality risks in pre-dialysis CKD [26,27] and HD patients [12,28]. On the other hand, the findings of large cohort studies on iron administration and mortality in HD patients have been controversial [28,29,30,31,32,33]. Miskulin et al. did not obtain consistent findings on the relationship between increasing cumulative IV iron doses over 1, 3, or 6 months and all-cause or cardiovascular mortality [30]. On the other hand, the Proactive IV Iron Therapy in HD Patients (PIVOTAL) study, involved a randomized controlled trial, which demonstrated that a high-dose intravenous iron regimen was superior to a low-dose regimen for HD patient outcomes and overall safety [34].

The KDIGO guidelines [2] do not recommend the intravenous administration of iron to patients with a serum ferritin levels consistently higher than 500 ng/mL, while this value is 300 ng/mL in the Japanese Society for Dialysis Therapy (JSDT) guidelines [3]. In the present study, only 25 patients (2.7%) had a serum ferritin levels ≥500 ng/mL, whereas 128 (13.6%) had a serum ferritin levels ≥300 ng/dL. Canavesse et al. used the superconducting quantum interference device method to quantify liver iron content relatively accurately in HD patients [35] and reported that patients with a serum ferritin levels ≥340 ng/mL had iron overload. Drüek et al. showed that internal carotid artery thickening worsened with increasing serum ferritin levels and iron dosages in HD patients receiving iron supplements [36], suggesting that iron administration or its accumulation in blood vessels leads to atherosclerosis in HD patients. Furthermore, excessive iron potentially triggers toxicity, such as oxidative stress and endothelial damage, in patients treated with intravenous iron, which may promote atherosclerosis and increase the risk of cardiovascular morbidity and mortality in CKD patients treated with or without HD [37,38,39].

In the present study, we evaluated and quantified functional iron deficiency by using the IS/TBI ratio and found that a large IS/TBI ratio was associated with a poor prognosis. Functional iron deficiency is characterized by the decreased availability of iron despite adequate IS due to the sequestration of iron within storage sites. This is commonly observed in patients with CKD and elevated C-reactive protein levels and is indicated by the coexistence of normal or high serum ferritin levels and low TSAT. A poor prognosis in CKD patients with functional iron deficiency has been reported [12,13,40]. However, there is currently no clear gold standard for assessing functional iron deficiency; therefore, its accurate diagnosis remains difficult [41]. In the present study, ROC curve analyses identified TBI and IS/TBI as more accurate predictors of mortality than serum ferritin levels and TSAT. Furthermore, the poor prognosis of the group with high IS/TBI levels suggested that improvement should be sought for patients with functional iron deficiency. For example, hypoxia-inducible factor prolyl-hydroxylase inhibitors have been reported to potentially improve functional iron deficiency [42].

Hepcidin is a pathogenic factor in most systemic iron disorders. Patients with CKD are a typical example of groups with markedly elevated hepcidin levels [43] due to the inflammatory milieu that accompanies uremia. However, as data on serum hepcidin levels were unavailable in the present study, the relationship of hepcidin with the IS/TBI ratio status in HD patients currently remains unclear.

The present study had several strengths. The quantification of TBI was associated with the prognosis of patients, suggesting the potential of TBI as a new clinical indicator to guide iron administration. Furthermore, although there has been no golden standard for functional iron deficiency, using the ratio of hemoglobin iron to TBI of IS may facilitate an accurate quantification. However, several limitations may have affected the generalizability of the present results. We did not have a control group, and the observational design allowed for only limited conclusions. Moreover, we could not prove a cause-and-effect relationship because of the study design. In addition, TBI values and other laboratory data were only measured at baseline; therefore, we were unable to examine the effects of changes from baseline during the follow-up using time-dependent analyses. Another limitation is that the TBI estimates used in the present study were for the general population in the US, and it remains unclear whether these results may be accurately extrapolated to Japanese HD patients. Furthermore, anemia in chronic disease may result in higher serum ferritin, which may change the TBI formula. However, no significant difference in the distribution of iron for hemoglobin and IS is expected between the general population and HD patients. In the present study, 39% of patients were excluded due to missing data on ferritin, which possibly resulted in a selection bias. As excessively high ferritin and hemoglobin levels were previously reported to be associated with a poor prognosis [16,44,45,46], excessively high TBI is expected to worsen the prognosis of patients; however, the upper limit of the optimal range of TBI was not identified in the present study.

## 5. Conclusions

In conclusion, the assessment of TBI represents a novel approach to examining iron metabolism and the relationship between TBI and mortality in patients on maintenance HD. Further studies are recommended to assess whether iron supplementation therapy that avoids low TBI, and the correction of an abnormal iron status will improve the survival of HD patients.

## Figures and Tables

**Table 1:** Baseline characteristics of the participants.

	All	TBI < 1.6 g	TBI 1.6–2.0 g	TBI ≥ 2.0 g	*p*-Value *
n	943	314	314	315	
Age (years)	68 (59–76)	71 (61–80)	69 (60–77)	64 (57–73)	<0.001
Female, n (%)	399 (42.3)	227 (72.3)	112 (35.7)	60 (19.0)	<0.001
Duration of HD (months)	83 (38–149)	97 (43–176)	84 (41–153)	62 (34–116)	<0.001
Pre-HD SBP (mmHg)	155 (142–166)	154 (141–166)	155 (144–166)	155 (141–167)	0.404
Pre-HD DBP (mmHg)	77 (71–83)	76 (70–82)	77 (71–83)	79 (72–86)	0.001
Dry weight (kg)	53.1 ± 10.7	43.3 ± 6.9	52.8 ± 6.9	62.7 ± 8.5	<0.001
Inter-dialysis weight gain (%)	4.5 (3.6–5.7)	4.7 (3.6–5.9)	4.6 (3.6–5.6)	4.3 (3.5–5.4)	0.009
Vascular access (AVF), n (%)	860 (91.2)	280 (89.2)	284 (90.4)	296 (94.0)	0.089
Diabetes mellitus, n (%)	291 (30.9)	81 (25.8)	93 (29.6)	117 (37.1)	0.007
History of CVD, n (%)	227 (24.1)	80 (25.5)	70 (22.3)	77 (24.4)	0.635
Walk independently, n (%)	760 (80.6)	227 (72.3)	252 (80.3)	281 (89.2)	<0.001
Smokers, n (%)	144 (15.3)	26 (8.3)	47 (15.0)	71 (22.5)	<0.001
Medications					
ESA use, n (%)	897 (95.1)	302 (96.2)	296 (94.3)	299 (94.9)	0.528
Iron supplement use, n (%)	737 (78.2)	239 (76.1)	247 (78.7)	251 (79.7)	0.537
Antihypertensive drug use, n (%)	731 (77.5)	229 (72.9)	254 (80.9)	248 (78.7)	0.047
-ARB use, n (%)	502 (53.2)	161 (51.3)	168 (53.5)	173 (54.9)	0.653
-ACEI use, n (%)	92 (9.8)	22 (7.0)	34 (10.8)	19 (6.0)	0.128
Laboratory data					
Hemoglobin (g/dL)	10.8 (10.0–11.5)	10.3 (9.6–11.0)	10.8 (10.2–11.5)	11.2 (10.6–11.8)	<0.001
TSAT (%)	23.7 (17.8–31.2)	22.2 (16.9–29.6)	25.0 (17.8–32.9)	24.0 (18.7–32.2)	0.047
Serum ferritin (ng/dL)	96.7 (42.5–205.1)	52.1 (27.4–132.9)	95.5 (42.0–193.2)	157 (74.0–273.7)	<0.001
Heme iron (mg)	1362 (1151–1586)	1069 (938–1202)	1353 (1238–1482)	1638 (1524–1814)	<0.001
Iron stores (mg)	490 (328–626)	323 (216–430)	492 (363–577)	682 (552–786)	<0.001
Total body iron (g)	1.8 (1.5–2.2)	1.4 (1.3–1.5)	1.8 (1.7–1.9)	2.3 (2.2–2.5)	<0.001
ERI	6.87 (4.62–11.34)	9.11 (6.47–15.38)	6.31 (4.32–10.50)	5.53 (3.55–7.30)	<0.001
Serum BUN (mg/dL)	66.3 ± 15.6	63.8 ± 15.7	66.5 ± 15.2	68.7 ± 15.5	<0.001
Serum creatinine (mg/dL)	10.8 ± 2.8	9.4 ± 2.1	10.9 ± 2.7	12.1 ± 2.9	<0.001
Serum adjusted Ca (mg/dL)	9.3 (8.9–9.7)	9.3 (8.9–9.7)	9.2 (8.8–9.6)	9.2 (8.9–9.7)	0.205
Serum phosphate (mg/dL)	5.1 (4.4–5.9)	5.1 (4.3–5.8)	5.1 (4.4–6.0)	5.3 (4.5–6.1)	0.154
Serum iPTH (mg/dL)	158 (81–277)	121 (58–214)	153 (81–280)	152 (85–250)	0.036
Serum albumin (g/dL)	3.8 (3.6–4.1)	3.7 (3.5–4.0)	3.8 (3.6–4.1)	3.9 (3.7–4.1)	<0.001
Serum CRP (mg/dL)	0.17 (0.06–0.70)	0.12 (0.05–0.58)	0.13 (0.05–0.67)	0.15 (0.08–0.70)	0.121
Single-pool Kt/V	1.18 ± 0.21	1.29 ± 0.21	1.17 ± 0.20	1.08 ± 0.16	<0.001

Normally distributed continuous data are reported as mean ± standard deviation and median (interquartile range) for non-normally distributed data. Categorical data are reported using frequency and percentage. *, one-way analysis of variance, Kruskal–Wallis test, or χ^2^ test. HD, hemodialysis; SBP, systolic blood pressure; DBP, diastolic blood pressure; AVF, arteriovenous fistula; CVD, cardiovascular disease; ESA, erythropoiesis-stimulating agent; ARB, angiotensin-receptor blocker; ACEI, angiotensin-converting enzyme inhibitor; TSAT, transferrin saturation; ERI, Erythropoietin Resistance Index; BUN, blood urine nitrogen; Ca, calcium; iPTH, intact parathyroid hormone; CRP, C-reactive protein.

**Table 2:** Relationship between baseline total body iron and the hazard ratios of all-cause, cardiovascular, infective, and other mortality.

TBI	Number of Deaths, Per 100 Patient Years	Unadjusted Model	Adjusted Mode l ^†^	Adjusted Model 2 ^‡^
All-cause				
T1	109, 9.6	2.70 (1.91–3.80)	2.51 (1.72–3.67)	1.87 (1.13–2.84)
T2	76, 6.1	1.70 (1.18–2.44)	1.50 (1.04–2.17)	1.18 (0.76–1.84)
T3	47, 3.6	1.00 (reference)	1.00 (reference)	1.00 (reference)
Cardiovascular				
T1	42, 3.7	1.75 (1.09–2.83)	1.64 (0.96–2.82)	1.31 (0.63–2.71)
T2	33, 2.7	1.24 (0.75–2.05)	1.12 (0.67–1.86)	1.00 (0.58–1.85)
T3	28, 2.1	1.00 (reference)	1.00 (reference)	1.00 (reference)
Infection				
T1	26, 2.3	5.09 (2.10–12.37)	4.22 (1.62–10.97)	2.05 (0.58–7.30)
T2	20, 1.6	3.51 (1.41–8.73)	2.94 (1.17–7.36	1.53 (0.48–4.85)
T3	6, 0.5	1.00 (reference)	1.00 (reference)	1.00 (reference)
Other				
T1	41, 3.6	3.61 (1.94–6.74)	3.61 (1.83–7.14)	2.59 (1.04–6.43)
T2	23, 1.9	1.84 (0.93–3.64)	1.66 (0.84–3.29)	1.44 (0.66–3.15)
T3	13, 1.0	1.00 (reference)	1.00 (reference)	1.00 (reference)

Values shown are hazard ratios (95% confidence intervals). T1: <1.6 g, T2: 1.6–2.0 g, T3: ≥2.0 g. ^†^ adjusted for age and sex. ^‡^ adjusted for age, sex, dry weight, walking independently, smokers, systolic and diastolic blood pressure, time of dialysis therapy, type of vascular access, diabetes mellitus, history of cardiovascular diseases, single-pool Kt/V, hemoglobin, serum albumin, serum C-reactive protein, adjusted serum phosphate and calcium, and the use of erythropoiesis-stimulating agents, angiotensin-receptor blockers, and angiotensin-converting enzyme inhibitors.

**Table 3:** Relationship between the baseline IS/TBI ratio and the hazard ratios of all-cause, cardiovascular, infective, and other mortality.

IS/TBI Ratio	Number of Deaths, Per 100 Patient Years	Unadjusted Model	Adjusted Model ^†^	Adjusted Model 2 ^‡^
All-cause				
T1	48, 4.0	1.00 (reference)	1.00 (reference)	1.00 (reference)
T2	73, 6.0	1.48 (1.03–2.12)	1.06 (0.73–1.53)	1.17 (0.79–1.73)
T3	111, 8.8	2.17 (1.54–3.04)	1.49 (1.05–2.10)	1.50 (1.03–2.18)
Cardiovascular				
T1	24, 2.0	1.00 (reference)	1.00 (reference)	1.00 (reference)
T2	32, 2.6	1.30 (0.77–2.21)	1.01 (0.58–1.79)	1.07 (0.60–1.90)
T3	47, 3.7	1.85 (1.13–3.02)	1.35 (0.82–2.23)	1.45 (0.83–2.53)
Infection				
T1	8, 0.7	1.00 (reference)	1.00 (reference)	1.00 (reference)
T2	19, 1.6	2.31 (1.01–5.29)	1.47 (0.64–3.39)	1.43 (0.56–3.63)
T3	25, 2.0	2.95 (1.33–6.54)	1.76 (0.78–3.95)	1.57 (0.64–3.86)
Other				
T1	16, 1.3	1.00 (reference)	1.00 (reference)	1.00 (reference)
T2	22, 1.8	1.32 (0.69–2.52)	0.95 (0.50–1.82)	1.24 (0.62–2.47)
T3	39, 3.1	2.52 (1.26–4.03)	1.55 (0.85–2.81)	1.69 (0.89–3.22)

Values shown are hazard ratios (95% confidence intervals). T1: <0.22 g, T2: 0.22–0.29 g, T3: ≥0.30 g. ^†^ adjusted for age and sex. ^‡^ adjusted for age, sex, dry weight, walking independently, smokers, systolic and diastolic blood pressure, time of dialysis therapy, type of vascular access, diabetes mellitus, history of cardiovascular diseases, single-pool Kt/V, hemoglobin, serum albumin, serum C-reactive protein, adjusted serum phosphate and calcium, and the use of erythropoiesis-stimulating agents, angiotensin-receptor blockers, and angiotensin-converting enzyme inhibitors.

**Table 4:** Subgroup analyses of TBI and all-cause mortality.

	Subgroups	No. of Patients	All-Cause Mortality, aHR (95% CI)	*p*-Value	*p* for Interaction *
Age, years	<68	491	1.39 (0.47–4.12)	0.557	0.070
	≥68	452	1.70 (0.92–3.12)	0.089	
Sex	Male	544	1.87 (0.98–3.57)	0.057	0.788
	Female	399	1.17 (0.31–2.55)	0.889	
Duration of HD, months	<83	470	1.72 (0.83–3.58)	0.147	0.420
	≥83	473	2.01 (0.93–4.36)	0.077	
Diabetes mellitus	Yes	291	1.26 (0.31–2.74)	0.499	0.192
	No	652	3.04 (1.51–6.14)	0.002	
Kt/V	<1.17	472	1.46 (0.74–2.89)	0.278	0.056
	≥1.17	471	2.25 (0.85–5.94)	0.101	
TSAT, %	<23.7	472	1.30 (0.41–4.09)	0.652	0.091
	≥23.7	471	2.58 (0.97–6.85)	0.058	
Alb, g/dL	<3.8	399	2.49 (1.28–4.84)	0.007	0.360
	>3.8	544	1.04 (0.52–2.07)	0.913	
CRP, mg/dL	<0.17	469	1.74 (0.72–4.18)	0.219	0.096
	≥0.17	474	2.22(1.15–4.32)	0.018	

The adjusted hazard ratio (aHR) represents mortality in the highest versus lowest tertile of TBI and was adjusted for potential confounders, such as age, sex, dry weight, walking independently, smokers, systolic and diastolic blood pressure, time of dialysis therapy, type of vascular access, diabetes mellitus, history of cardiovascular diseases, single-pool Kt/V, hemoglobin, serum albumin, serum C-reactive protein, adjusted serum phosphate and calcium, and the use of erythropoiesis-stimulating agents, angiotensin-receptor blockers, and angiotensin-converting enzyme inhibitors. HRs in the highest TBI tertile were elevated across the subgroups. * *p* for interaction indicates significance between both subgroups in each TBI strata.

**Table 5:** Subgroup analyses of the IS/TBI ratio and all-cause mortality.

	Subgroups	No. of Patients	All-Cause Mortality, aHR (95% CI)	*p*-Value	*p* for Interaction *
Age, years	<68	491	2.32 (1.10–4.90)	0.027	0.077
	≥68	452	1.36 (0.86–2.16)	0.185	
Sex	Male	544	1.66 (0.87–3.16)	0.122	0.936
	Female	399	1.55 (0.93–2.58)	0.094	
Duration of HD, months	<83	470	1.48 (0.88–2.49)	0.136	0.409
	≥83	473	1.36 (0.74–2.49)	0.880	
Diabetes mellitus	Yes	291	2.14 (1.11–4.13)	0.023	0.711
	No	652	1.36 (0.84–2.21)	0.208	
Kt/V	<1.17	472	1.73 (1.01–2.96)	0.046	0.736
	≥1.17	471	1.60 (0.90–2.83)	0.109	
TSAT, %	<23.7	472	2.41 (1.25–4.63)	0.009	0.887
	≥23.7	471	1.23 (0.54–2.78)	0.626	
Alb, g/dL	<3.8	399	1.32 (0.79–2.20)	0.283	0.155
	>3.8	544	2.24 (1.23–4.09)	0.009	
CRP, mg/dL	<0.17	469	1.29 (0.71–2.36)	0.410	0.681
	≥0.17	474	1.36 (0.80–2.31)	0.255	

The adjusted hazard ratio (aHR) represents mortality in the highest versus lowest tertile of TBI and was adjusted for potential confounders, such as age, sex, dry weight, walking independently, smokers, systolic and diastolic blood pressure, time of dialysis therapy, type of vascular access, diabetes mellitus, history of cardiovascular diseases, single-pool Kt/V, hemoglobin, serum albumin, serum C-reactive protein, adjusted serum phosphate and calcium, and the use of erythropoiesis-stimulating agents, angiotensin-receptor blockers, and angiotensin-converting enzyme inhibitors. HRs in the highest TBI tertile were elevated across the subgroups. * *p* for interaction indicates significance between both subgroups in each IS/TBI strata.

## Data Availability

The data that support the findings of this study are available from the corresponding author, T.T., upon reasonable request.
